# Diathermy induced gas explosion during laparotomy: a case report

**DOI:** 10.1093/jscr/rjad460

**Published:** 2023-08-09

**Authors:** Martin Sindani, Paul William Itule Lugwaja, Novath Ngowi

**Affiliations:** Department of Surgery, Muhimbili University of Health and Allied Sciences (MUHAS), Dar es Salaam, Tanzania; Department of Surgery, Muhimbili University of Health and Allied Sciences (MUHAS), Dar es Salaam, Tanzania; Department of Surgery, Muhimbili University of Health and Allied Sciences (MUHAS), Dar es Salaam, Tanzania

**Keywords:** diathermy, laparotomy, explosion

## Abstract

Diathermy-induced explosions in the gastrointestinal tract can occur during laparotomy and can be fatal. Hydrogen and methane are the most common flammable gases that are produced in the gastrointestinal tract. A 35-year-old male was referred to a tertiary hospital with abdominal pain after being assaulted and stabbed in the abdomen. He presented with a 2-cm wound in the midline below the xiphoid process and with an omentum protruding through the wound. Explorative laparotomy was done to repair the jejunal perforation, but a gastric blast occurred, making more than half of the gastric pouch non-viable. The operation was converted to a near-total gastrectomy with Roux-en-Y gastrojejunostomy. During open abdominal surgery, fires and explosions can occur. Precautions should be taken during open abdominal surgery to reduce the risk of explosions, such as allowing combustible gases to exit the cavity before using diathermy.

## INTRODUCTION

Diathermy-induced explosions in the gastrointestinal tract are serious iatrogenic complications and can be fatal. Most of these explosions have occurred in the colon and rectum during endoscopy, but explosions have also occurred during colostomy [1], and a single case of jejunal gas explosion has been reported [2]. Also, rare cases of combustible eructation have been reported [3], but studies of the origin and composition of combustible gases in the stomach are lacking. The combustible gases hydrogen (H_2_) and methane (CH_4_) are produced by bacteria and are usually limited to the colon, but in individuals with bacterial overgrowth of the small bowel, gas production to some degree will also occur there [3]. The explosive range of hydrogen in air is 4–72% and that of methane is 5–15%, but neither gas is combustible unless oxygen concentrations of at least 5% are present [4].

Various measures have been proposed to reduce the risk of explosion during electrosurgical procedures in the large bowel: the infusion of CO_2_ through the scope [3, 5], the avoidance of mannitol [6] and oral antibiotics [3] in the preparation procedure before endoscopy and the avoidance of diathermy in the performance of colectomy [1]. Whether the gas was produced in the stomach itself or was transferred to the stomach by the perioperative retrograde milking of the jejunal contents, or both, is unknown. The common denominator for these explosions of the upper gastrointestinal tract is obstruction. In cases of gastrointestinal tract obstruction, the diathermy knife should not be used when entering the gastrointestinal lumen.

## CASE PRESENTATION

A 35-year-old male referral from a peripheral health center presented with abdominal pain for 1 day. He was assaulted by group of unknown people while riding his motorcycle on his way back home and sustained a penetrating abdominal injury, whereby he was stabbed in the abdomen and around the upper arm. The patient reports loss of consciousness following the event for an undetermined period of time until he found himself awake at the hospital, stained with blood and weak; he had no vomiting, no blood in his urine, no consumption of intoxicants before driving, no medical history of epilepsy and he was able to use all of his four limbs.

The patient’s last meal was ~4 hours before the occurrence of the event. He was referred to the tertiary hospital, where a formal abdominal ultrasound was done upon arrival at the emergency department and revealed significant fluid collection in both the splenorenal and hepatorenal recesses.

Upon general examination, he was pale with normal vital signs. On abdominal examination, patient had muscle guarding, 2-cm wound in the midline ~10 cm below the xiphoid process, gushing out air with every respiratory cycle, not actively oozing blood; omentum can be seen protruding through the wound with generalized abdominal tenderness on superficial and deep palpation and distant bowel sounds. Other systems were normal except for the musculoskeletal system, which had two superficial lacerations of ~1 cm each around the left deltoid muscle. We had the impression of a peritoneal violation and visceral injury, hence planned for explorative laparotomy.

**Figure 1:**
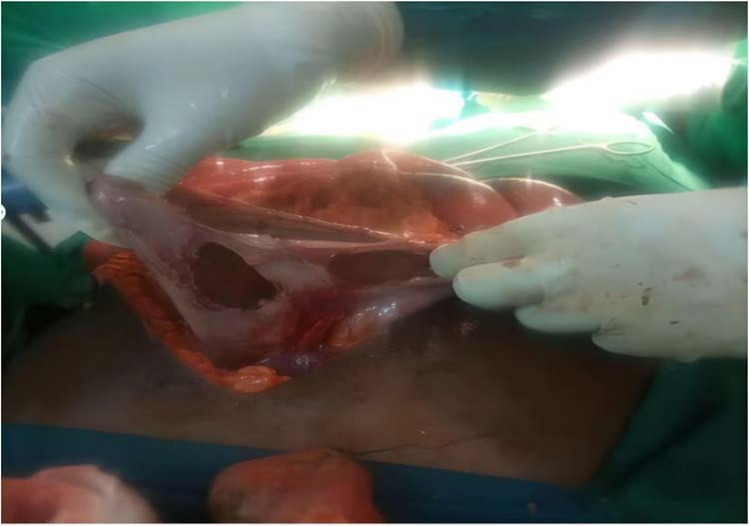
Gastric blast occurred intraoperative due explosion after gas–diathermy reaction.

His lab results were normal, and the patient was reviewed by the anesthesia team and was cleared for surgery. Upon explorative laparotomy, a penetrating wound crossing all the abdominal wall layers and violating the peritoneal space was revealed: hemo-peritoneum with small bowel contents in the peritoneal cavity mixed with food particles; omental laceration below its attachments to the transverse colon; mesenteric laceration with mesenteric border jejunal perforation with normal liver, spleen and other viscera. What was done was peritoneal lavage with warm normal saline, resection of the jejunal perforation site and end-to-end primary anastomosis intraoperatively. Upon small bowel resection, gastric blast occurred due to the gas–diathermy reaction, making more than half of the gastric pouch burnt and non-viable. The operation was converted to a near-total gastrectomy with Roux-en-Y gastrojejunostomy, as shown (Fig. 1) in the intraoperative image.

## DISCUSSION

Fires and explosions have occurred in the operating theater for many years. Flammable inhalation anesthetic agents were responsible for many incidents in the past, but these are no longer available in many countries. Other causes of fires and explosions still exist in the operating theater and, from time to time, result in serious and occasionally fatal injuries. Flammable gases are produced at all levels of the gastrointestinal tract. Hydrogen and methane are the most common flammable gases produced in the bowel. The proportions will vary with diet, digestion and metabolism [5]. The large bowel may contain 40% of flammable gases, with the maximum recorded concentrations being 69% hydrogen and 56% methane. Human flatus has been shown to contain 44% hydrogen and 30% methane [7].

Bowel preparation of the colon with mannitol or lactulose is associated with an increase in combustible gas, resulting in explosions during colonic resection caused by electrosurgery [2]. Oxygen concentrations in the normal gastrointestinal tract decrease from 10% in the stomach to 5% in the colon, but anesthesia using oxygen and nitrous oxide increases the concentration of both gases in the bowel. Hydrogen production, unlike methane, is dependent on the bacterial metabolism of ingested material; its production is also increased after preoperative administration of mannitol. Methane production is independent of all of these factors [8].

The explosive range of hydrogen is 4–72% and that of methane is 5–15% [5], but neither is combustible in <5% oxygen [1]. Fires and explosions are rare events, but they can be devastating both in terms of structural damage to equipment and theaters and to human lives.

## CONCLUSION

Rarely, during open abdominal surgery, do fires and explosions occur in the operating room. When they do happen, they have the potential to seriously endanger patients, hurt employees, damage theaters and equipment structurally . To limit the danger of electrosurgery-induced ignition, precautions should be taken during laparotomy.

## Data Availability

Not applicable.
